# Supplementary material for: Mechanism of nucleotide discrimination by the translesion synthesis polymerase Rev1
Source: Nat Commun. 2022 May 24;13:2876. doi: 10.1038/s41467-022-30577-0 (PMC9130138; doi:10.1038/s41467-022-30577-0)
Supplement: Supplementary file 1 — Supplementary Information [file 41467_2022_30577_MOESM1_ESM.pdf]

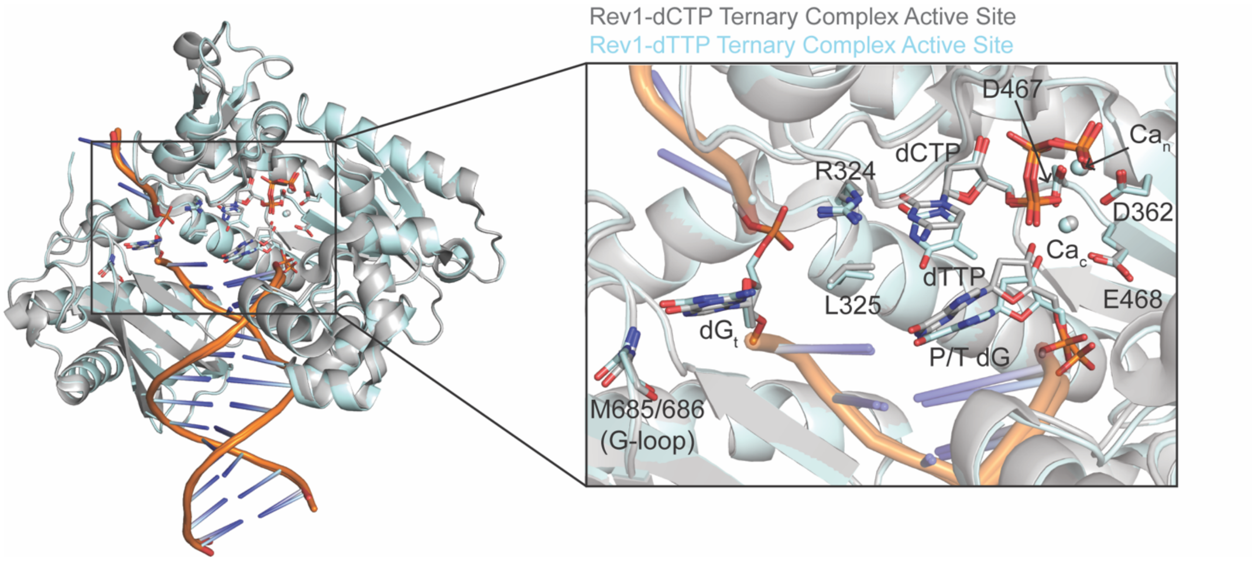

Supplementary Figure 1. Overall comparison of the Rev1/DNA/dTTP and Rev1/DNA/dCTP ternary complex structures. Comparison of the overall structure (left) and active site (right) of the Rev1/DNA/dTTP (blue) and Rev1/DNA/dCTP (grey) ternary complexes**.** Key protein and DNA residues are shown as sticks.

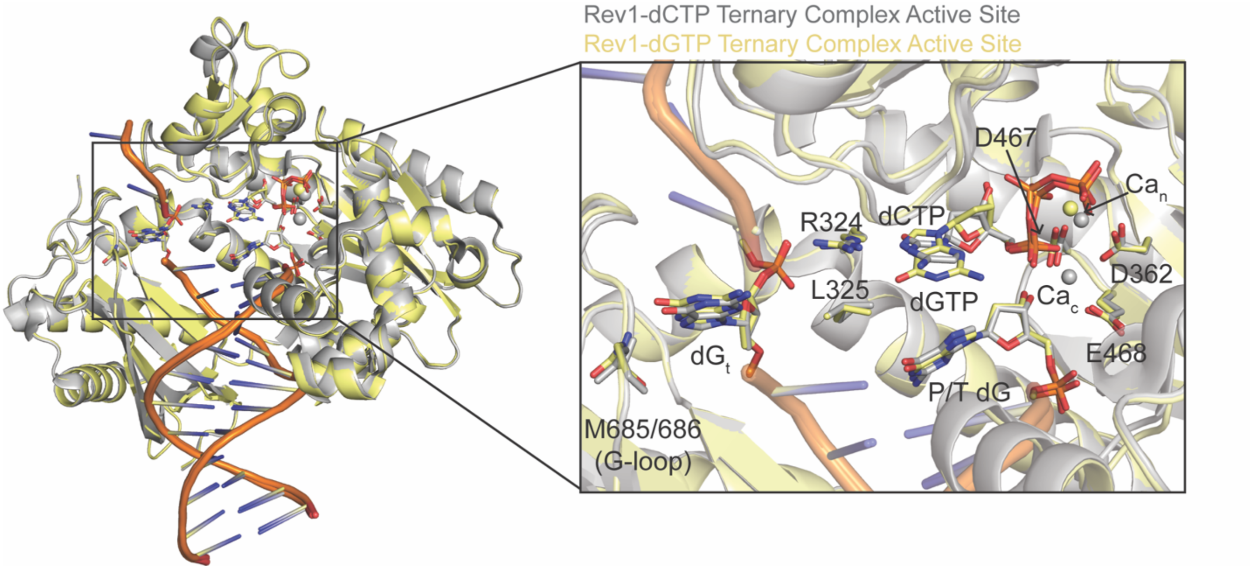

Supplementary Figure 2. Overall comparison of the Rev1/DNA/dGTP and Rev1/DNA/dCTP ternary complex structures. Comparison of the overall structure (left) and active site (right) of the Rev1/DNA/dGTP (yellow) and Rev1/DNA/dCTP (grey) ternary complexes**.** Key protein and DNA residues are shown as sticks.

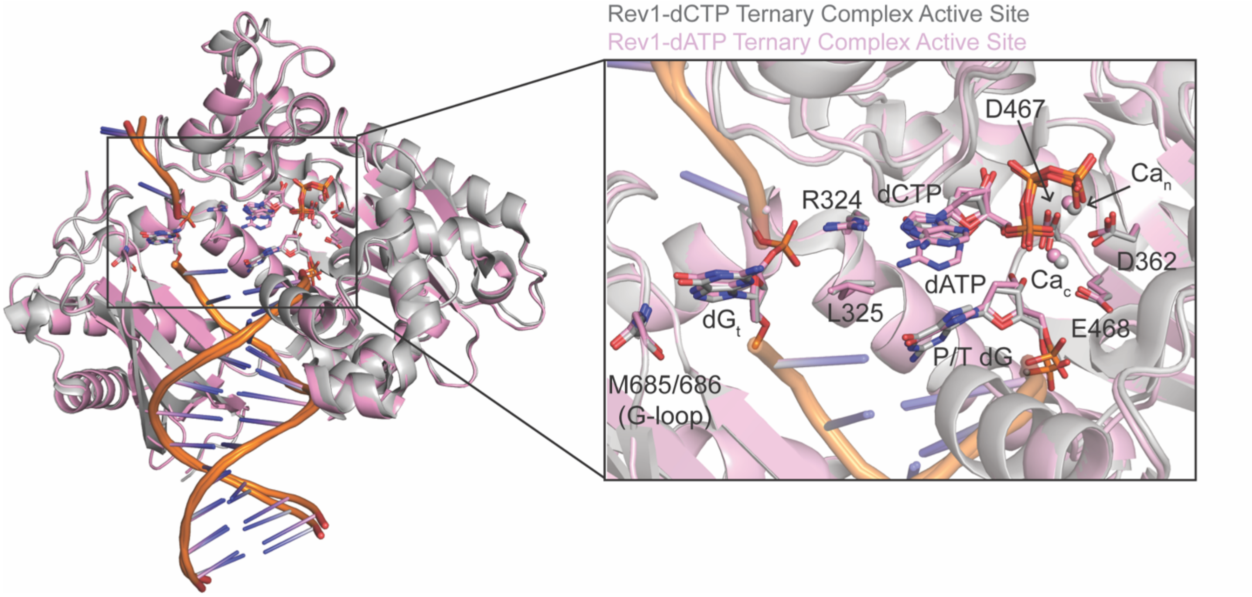

Supplementary Figure 3. Overall comparison of the Rev1/DNA/dATP and Rev1/DNA/dCTP ternary complex structures. Comparison of the overall structure (left) and active site (right) of the Rev1/DNA/dATP (pink) and Rev1/DNA/dCTP (grey) ternary complexes**.** Key protein and DNA residues are shown as sticks.

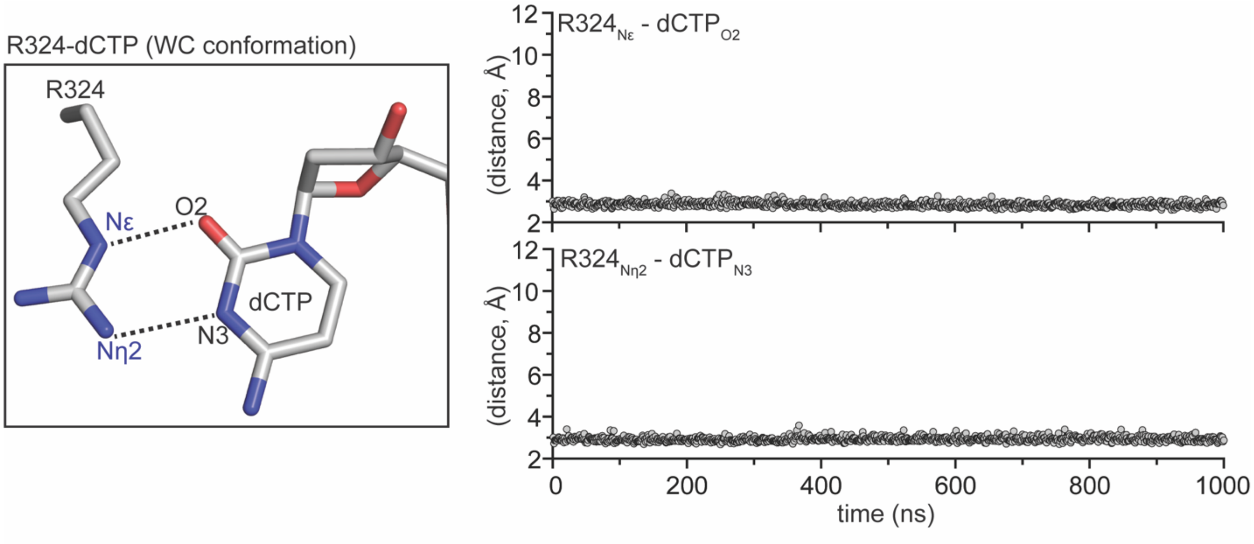

Supplementary Figure 4. Molecular dynamics simulation of Rev1/DNA/dCTP ternary complex structure. Focused view of the Rev1 R324 and incoming dCTP (Watson-Crick conformation) showing the distances monitored throughout the MD simulation (left). Distance profiles for $R324_{N\varepsilon}$-$dCTP_{O2}$ and the $R324_{N\eta2}$-$dCTP_{N3}$ in the Rev1/DNA/dCTP ternary complex simulation (right). Each datapoint represents the distance (Å) between the indicated atoms at a single snapshot (1ns) from the MD simulation. This figure is adapted from Weaver et al., (2020) Visualizing Rev1 catalyze protein-template DNA synthesis. *PNAS*, **117 (41)** 25494-25504.

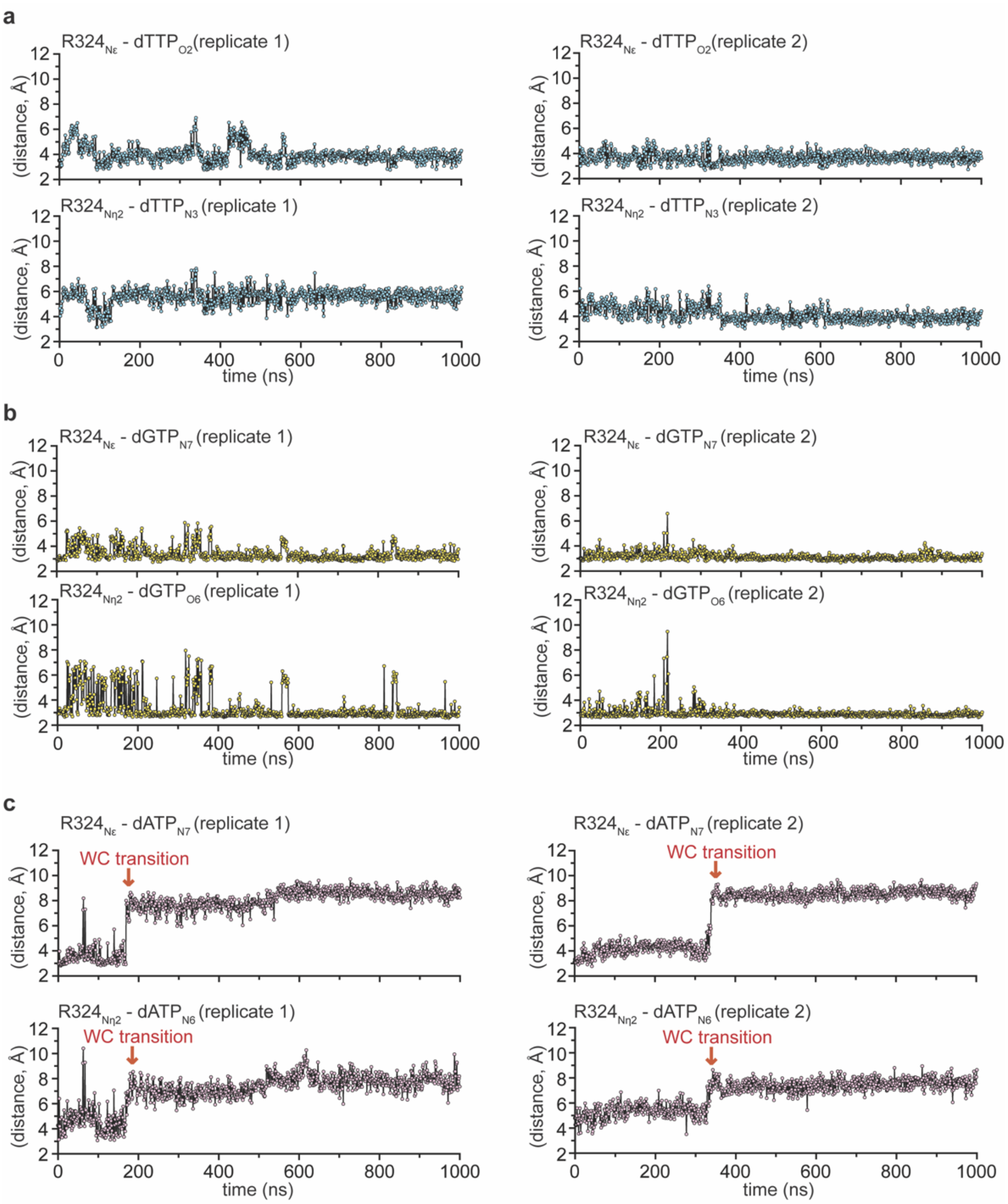

Supplementary Figure 5. Replicate molecular dynamics simulations of Rev1/DNA/dNTP ternary complex structures. (a) Distance profiles for R324$_{NE}$-dTTP$_{O2}$ and the R324$_{Nn2}$-dTTP$_{N3}$ in the Rev1/DNA/dTTP ternary complex replicate MD simulations. Each datapoint represents the distance (Å) between the indicated atoms at a single snapshot (1ns) from the MD simulation. **(b)** Distance profiles for R324$_{N\varepsilon}$-dGTPN7 and the R324$_{Nn2}$-dGTP$_{O6}$ in the Rev1/DNA/dGTP ternary complex replicate MD simulations. Each datapoint represents the distance (Å) between the indicated atoms at a single snapshot (1ns) from the MD simulation. **(c)** Distance profiles for R324$_{N\varepsilon}$-dATP$_{N7}$ and the R324$_{Nn2}$-dATP$_{N6}$ in the Rev1/DNA/dATP ternary complex replicate MD simulations. Each datapoint represents the distance (Å) between the indicated atoms at a single snapshot (1ns) from the MD simulation. The dATP transition from Hoogsteen to Watson-Crick conformation is denoted with a red arrow. Source data for this figure are provided as a Source Data file.

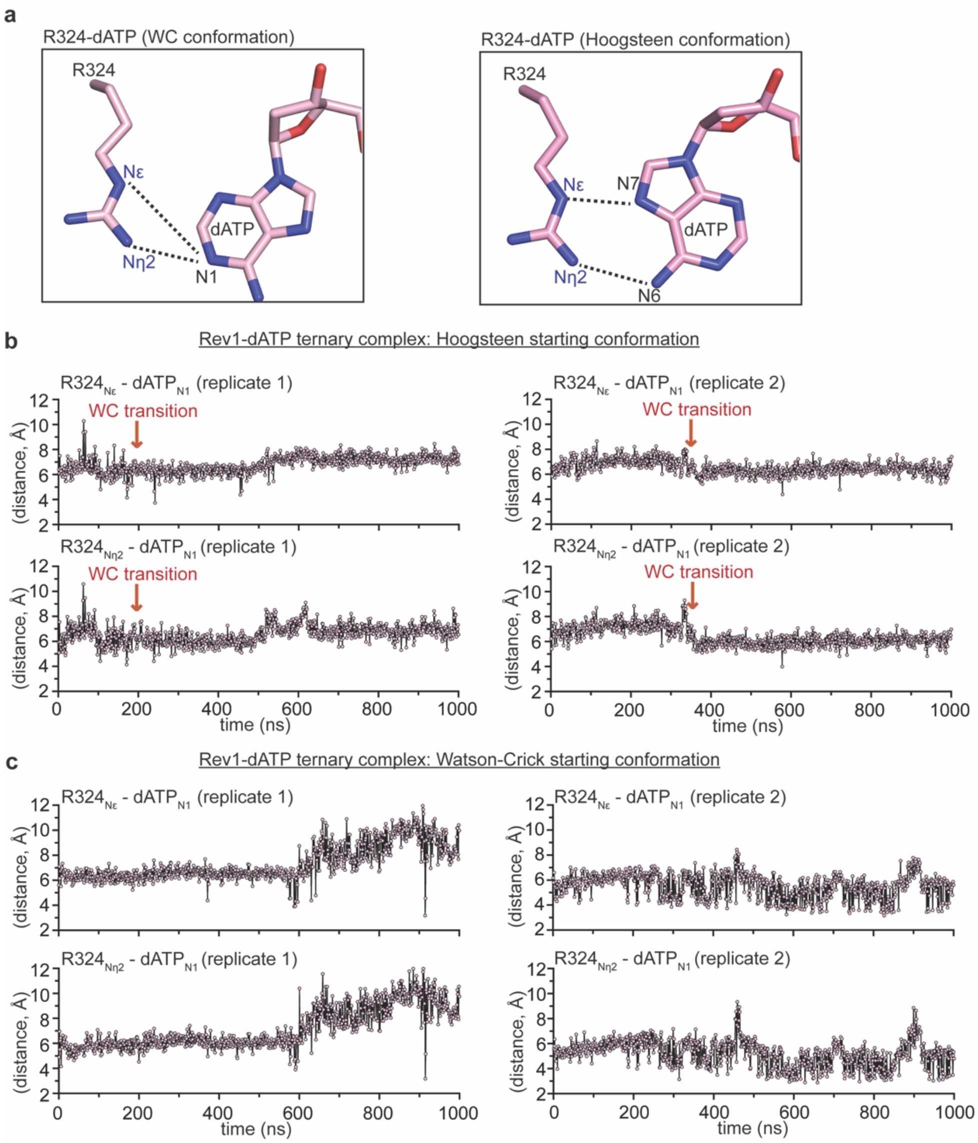

Supplementary Figure 6. Replicate molecular dynamics simulation of Rev1/DNA/dATP ternary complex structure. (a) Focused view of the Rev1 R324 and incoming dATP (Watson-Crick conformation) showing the distances monitored throughout the MD simulation (left). Focused view of the Rev1 R324 and incoming dATP (Hoogsteen conformation) for comparison (right). **(b)** Distance profiles for $R324_{N\varepsilon}$-$dATP_{N1}$ and the $R324_{N\eta 2}$-$dATP_{N1}$ in the Rev1/DNA/dATP ternary complex replicate MD simulations. Each datapoint represents the distance (Å) between the indicated atoms at a single snapshot (1ns) from the MD simulation. The MD simulations were started with the dATP in the Hoogsteen conformation. The dATP transition from Hoogsteen to Watson-Crick conformation is denoted with a red arrow. **(c)** Distance profiles for $R324_{N\varepsilon}$-$dATP_{N1}$ and the $R324_{N\eta 2}$-$dATP_{N1}$ in the Rev1/DNA/dATP ternary complex replicate MD simulations. Each datapoint represents the distance (Å) between the indicated atoms at a single snapshot (1ns) from the MD simulation. The MD simulations were started with the dATP in the theoretical Watson-Crick conformation. Source data for this figure are provided as a Source Data file.

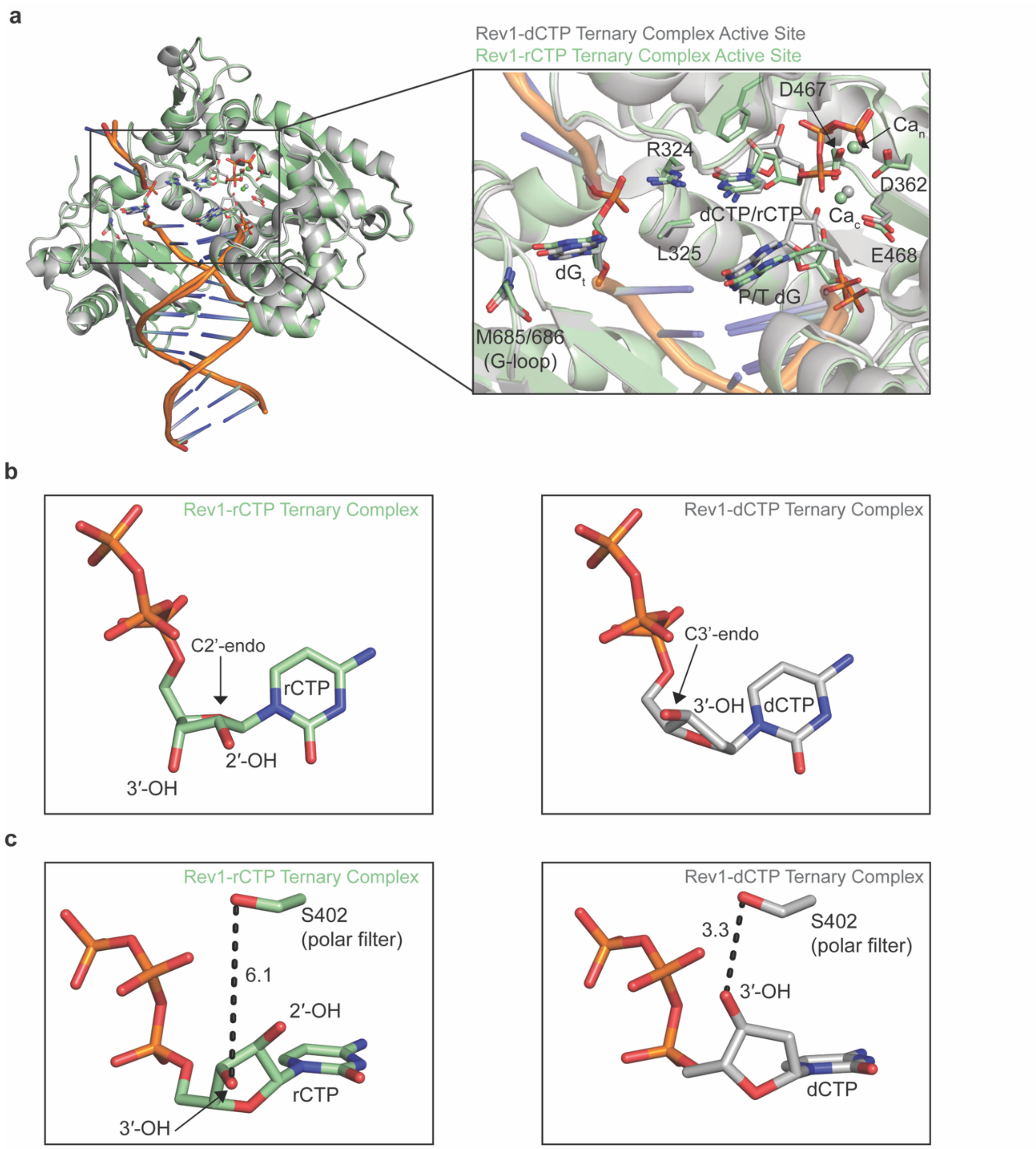

Supplementary Figure 7. Overall comparison of the Rev1/DNA/rCTP and Rev1/DNA/dCTP ternary complex structures. (a) Comparison of the overall structure (left) and active site (right) of the Rev1/DNA/rCTP (green) and Rev1/DNA/dCTP (grey) ternary complexes**.** Key protein and DNA residues are shown as sticks. **(b)** A focused view of the incoming nucleotide sugar pucker in the Rev1/DNA/rCTP (left) and Rev1/DNA/dCTP (right) ternary complex structures. **(c)** A focused view of the incoming nucleotide position and Rev1 S402 polar filter residue in the Rev1/DNA/rCTP (green sticks, left) and Rev1/DNA/dCTP (grey sticks, right) ternary complex structures. Key distances (Å) are shown as black dashed lines.

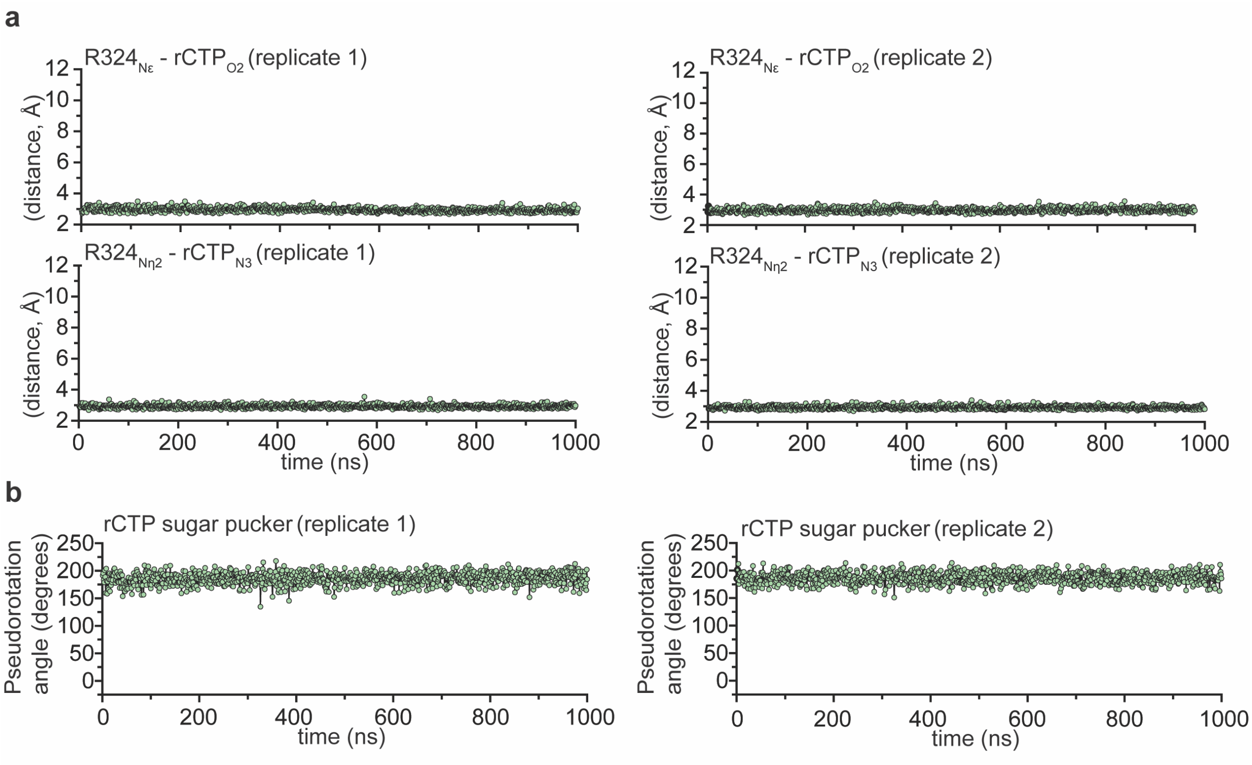

Supplementary Figure 8. Replicate molecular dynamics simulations of Rev1/DNA/rCTP ternary complex structure. (a) Distance profiles for $R324_{N\varepsilon}$-$rCTP_{O2}$ and the $R324_{N\eta 2}$-$rCTP_{N3}$ in the Rev1/DNA/rCTP ternary complex replicate MD simulations. Each datapoint represents the distance (Å) between the indicated atoms at a single snapshot (1ns) from the MD simulation. **(b)** Pseudorotation angle profile for the rCTP ribose sugar throughout the replicate MD simulations. Each datapoint represents the pseudorotation angle of the rCTP ribose sugar at a single snapshot (1ns) from the MD simulation. Source data for this figure are provided as a Source Data file.

Supplementary Table 1: Rev1 pre-steady state kinetics

Organism	NTP	k_{pol}	K_d (NTP)	F_{rel}[1]
H. sapien[3]	dCTP	22.4 ± 0.9	2.2 ± 0.3	-
	dTTP	0.88 ± 0.06	22.7 ± 7	260
	dGTP	6.3 ± 0.3	90 ± 10	140
	dATP	0.050 ± 0.004	70 ± 20	14000
	rCTP	0.098 ± 0.002	2.7 ± 0.2	280
S. cerevisiae[4]	dCTP	18 ± 1	32 ± 7	--
	dTTP	0.20 ± 0.02	700 ± 100	2000
	dGTP	0.97 ± .0.04	460 ± 40	270
	dATP	ND[2]	ND[2]	ND[2]

[1]F_{rel} is the relative catalytic efficiency of incorporating the given dNTP versus dCTP, which is kpol(dCTP)/kpol(dNTP) times K_d (dNTP)/K_d (dCTP).
[2]ND is not detected.
[3]Values from Brown, J.A., Fowler, J.D. and Suo, Z. (2010) Kinetic basis of nucleotide selection employed by a protein template-dependent DNA polymerase. *Biochemistry*, **49**, 5504-5510.
[4]Values from Pryor, J.M. and Washington, M.T. (2011) Pre-steady state kinetic studies show that an abasic site is a cognate lesion for the yeast Rev1 protein. *DNA repair*, **10**, 1138-1144.

Supplementary Table 2: Data collection and refinement statistics for Rev1 crystal structures

	Rev1/DNA/dTTP Ternary Complex	Rev1/DNA/dGTP Ternary Complex	Rev1/DNA/dATP Ternary Complex	Rev1/DNA/rCTP Ternary Complex
**Data collection**				
Space group	$P2_12_12_1$	$P2_12_12_1$	$P2_12_12_1$	$P2_12_12_1$
Cell dimensions				
a, *b*, *c* (Å)	62.2,73.6,117.8	62.2,73.6,117.0	62.1,73.3,115.4	62.2,73.5,117.7
α, β, γ (°)	90,90,90	90,90,90	90,90,90	90,90,90
Resolution (Å)	25 – 1.70	25 – 2.03	25 – 1.80	25 – 1.75
R_{meas}^{a} (%)	0.078 (>1)	0.165 (>1)	0.077 (0.924)	0.084 (.952)
$I/\sigma I$	15.1 (1.3)	6.9 (5.0)	12.5 (0.7)	11.5 (0.81)
cc1/2[b]	0.529	0.443	0.556	0.542
Completeness[a] (%)	99.8 (99.6)	99.5 (99.2)	99.3 (98.6)	98.1 (94.4)
Redundancy[a]	4.3 (3.0)	4.4 (3.0)	3.5 (2.4)	2.5 (1.7)
**Refinement**				
Resolution (Å)	24.66 – 1.70	24.59 – 2.01	24.65 – 1.81	24.63 – 1.75
No. reflections	58143	24002	55331	77480
$R_{work/}$ R_{free}	18.23/22.53	18.12/24.22	19.31/22.96	18.29/22.81
No. atoms				
Protein	3527	3570	3574	3551
DNA	571	568	569	571
Water	626	212	306	358
B-factors ($Å^2$)				
Protein	18.52	32.13	24.96	22.43
DNA	25.65	40.97	38.45	31.60
Water	30.92	35.35	31.00	31.59
R.m.s deviations				
Bond length (Å)	0.008	0.009	0.013	0.009
Bond angles (º)	0.991	1.053	1.278	1.055
PDB ID	7T18	7T19	7T1A	7T1B

[a] Highest resolution shell is shown in parenthesis
[b] Highest resolution shell only.
