# Supplementary material for: Mechanism of nucleotide discrimination by the translesion synthesis polymerase Rev1
Source: Nat Commun. 2022 May 24;13:2876. doi: 10.1038/s41467-022-30577-0 (PMC9130138; doi:10.1038/s41467-022-30577-0)
Supplement: Supplementary file 2 — Reporting Summary [file 41467_2022_30577_MOESM2_ESM.pdf]

nature portfolio

Corresponding author(s): Bret Freudenthal

Last updated by author(s): May 1, 2022

Reporting Summary

Nature Portfolio wishes to improve the reproducibility of the work that we publish. This form provides structure for consistency and transparency in reporting. For further information on Nature Portfolio policies, see our Editorial Policies and the Editorial Policy Checklist.

Statistics

For all statistical analyses, confirm that the following items are present in the figure legend, table legend, main text, or Methods section.

n/a	Confirmed	
☒	☐	The exact sample size (*n*) for each experimental group/condition, given as a discrete number and unit of measurement
☒	☐	A statement on whether measurements were taken from distinct samples or whether the same sample was measured repeatedly
☒	☐	The statistical test(s) used AND whether they are one- or two-sided<br>*Only common tests should be described solely by name; describe more complex techniques in the Methods section.*
☒	☐	A description of all covariates tested
☒	☐	A description of any assumptions or corrections, such as tests of normality and adjustment for multiple comparisons
☒	☐	A full description of the statistical parameters including central tendency (e.g. means) or other basic estimates (e.g. regression coefficient) AND variation (e.g. standard deviation) or associated estimates of uncertainty (e.g. confidence intervals)
☒	☐	For null hypothesis testing, the test statistic (e.g. *F*, *t*, *r*) with confidence intervals, effect sizes, degrees of freedom and *P* value noted<br>*Give P values as exact values whenever suitable.*
☒	☐	For Bayesian analysis, information on the choice of priors and Markov chain Monte Carlo settings
☒	☐	For hierarchical and complex designs, identification of the appropriate level for tests and full reporting of outcomes
☒	☐	Estimates of effect sizes (e.g. Cohen's *d*, Pearson's *r*), indicating how they were calculated

Our web collection on statistics for biologists contains articles on many of the points above.

Software and code

Policy information about availability of computer code

Data collection	All X-ray crystallography data was collected with HKL3000R (v705c) using a Rigaku MicroMax-007 HF rotating anode diffractometer . All molecular dynamics data were generated using AMBER (v16) suite of programs for biomolecular simulations with AMBER's ff14SB 64 force-fields and simple point change (SPC) water model.
Data analysis	All X-ray crystallography data was indexed and scaled using HKL3000R(v705c). All model building and refinement for X-ray crystallography was performed using PHENIX (1.19.2-4158) and Coot (0.9.5). All figures containing structures in this manuscript were generated using PyMol (2.3.2). The sugar puckers for incoming NTPs were determined by calculating pseudorotation angles using the web 3DNA (2.0) server.

For manuscripts utilizing custom algorithms or software that are central to the research but not yet described in published literature, software must be made available to editors and reviewers. We strongly encourage code deposition in a community repository (e.g. GitHub). See the Nature Portfolio guidelines for submitting code & software for further information.

Data

Policy information about availability of data

All manuscripts must include a data availability statement. This statement should provide the following information, where applicable:

- Accession codes, unique identifiers, or web links for publicly available datasets
- A description of any restrictions on data availability
- For clinical datasets or third party data, please ensure that the statement adheres to our policy

Atomic coordinates and structure factors for the crystal structures reported in this manuscript have been deposited with the Protein Data bank under accession numbers 7T18 [https://doi.org/10.2210/pdb7T18/pdb], 7T19 [https://doi.org/10.2210/pdb7T19/pdb], 7T1A [https://doi.org/10.2210/pdb7T1A/pdb], and 7T1B [https://doi.org/10.2210/pdb7T1B/pdb]. Atomic coordinates and structure factors for the previously determined Rev1-dCTP ternary complex structure were

accessed from the Protein Data bank under accession number 6X6Z [https://doi.org/10.2210/pdb6X6Z/pdb]. All molecular dynamics simulation trajectories have been deposited in the Data Archiving and Networking Services (DANS) repository and are available at https://doi.org/10.17026/dans-xaw-36b2. Source data are provided with this paper.

Field-specific reporting

Please select the one below that is the best fit for your research. If you are not sure, read the appropriate sections before making your selection.

☒ Life sciences ☐ Behavioural & social sciences ☐ Ecological, evolutionary & environmental sciences

For a reference copy of the document with all sections, see nature.com/documents/nr-reporting-summary-flat.pdf

Life sciences study design

All studies must disclose on these points even when the disclosure is negative.

Sample size	Sample size were predetermined based on accepted standards within the field, and not predetermined using statistical methods.
Data exclusions	For X-ray crystallography, data with a CC1/2 of less than 0.4 were excluded, which is within the field standard. No data was excluded from the MD simulations.
Replication	All MD simulations were performed as two independent replicate experiments to ensure reproducibility of the data. No replicate experiments were performed for X-ray crystallography as is the accepted standard within the field.
Randomization	For X-ray crystallography, a random subset of ~10% of reflections for each structure were set aside for calculating Rfree values. Randomization was not performed for the MD simulations.
Blinding	None, blinding was not performed in these studies. Blinding was not feasible because the experimenters required prior knowledge of which incoming nucleotide was soaked into the crystals in order to build the models for X-ray crystallography, and to correctly extract parameters for each different Rev1-NTP ternary complexes for the MD simulations.

Reporting for specific materials, systems and methods

We require information from authors about some types of materials, experimental systems and methods used in many studies. Here, indicate whether each material, system or method listed is relevant to your study. If you are not sure if a list item applies to your research, read the appropriate section before selecting a response.

Materials & experimental systems

n/a	Involved in the study
☒	☐ Antibodies
☒	☐ Eukaryotic cell lines
☒	☐ Palaeontology and archaeology
☒	☐ Animals and other organisms
☒	☐ Human research participants
☒	☐ Clinical data
☒	☐ Dual use research of concern

Methods

n/a	Involved in the study
☒	☐ ChIP-seq
☒	☐ Flow cytometry
☒	☐ MRI-based neuroimaging
